# Photo-Responsive Graphene and Carbon Nanotubes to Control and Tackle Biological Systems

**DOI:** 10.3389/fchem.2018.00102

**Published:** 2018-04-12

**Authors:** Francesca Cardano, Marco Frasconi, Silvia Giordani

**Affiliations:** ^1^Nano Carbon Materials, Istituto Italiano di Tecnologia, Turin, Italy; ^2^Department of Chemistry and Industrial Chemistry, University of Genoa, Genoa, Italy; ^3^Department of Chemical Sciences, University of Padova, Padova, Italy; ^4^Department of Chemistry, University of Turin, Turin, Italy

**Keywords:** photochromism, carbon nanomaterials, azobenzene, spiropyran, drug delivery, bioimaging

## Abstract

Photo-responsive multifunctional nanomaterials are receiving considerable attention for biological applications because of their unique properties. The functionalization of the surface of carbon nanotubes (CNTs) and graphene, among other carbon based nanomaterials, with molecular switches that exhibit reversible transformations between two or more isomers in response to different kind of external stimuli, such as electromagnetic radiation, temperature and pH, has allowed the control of the optical and electrical properties of the nanomaterial. Light-controlled molecular switches, such as azobenzene and spiropyran, have attracted a lot of attention for nanomaterial's functionalization because of the remote modulation of their physicochemical properties using light stimulus. The enhanced properties of the hybrid materials obtained from the coupling of carbon based nanomaterials with light-responsive switches has enabled the fabrication of smart devices for various biological applications, including drug delivery, bioimaging and nanobiosensors. In this review, we highlight the properties of photo-responsive carbon nanomaterials obtained by the conjugation of CNTs and graphene with azobenzenes and spiropyrans molecules to investigate biological systems, devising possible future directions in the field.

## Introduction

**Figure 1:**
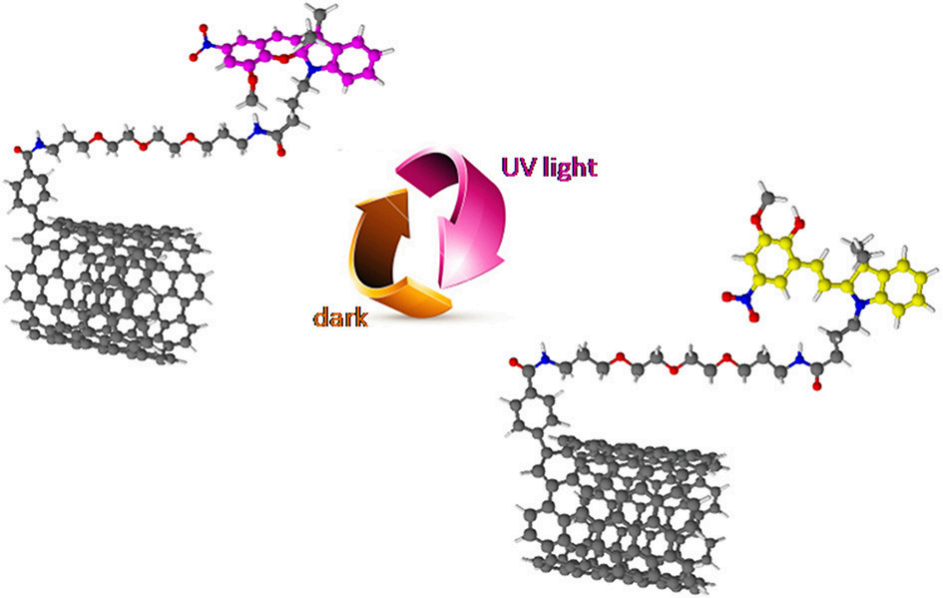
A schematic representation of a light-responsive carbon based material: CNTs functionalized with SPs. Reproduced with permission from Del Canto et al. (2012) with permission of the PCCP Owner Societies.

Carbon nanomaterials, such as carbon nanotubes (CNTs) and graphene, present unique physicochemical properties and have been studied as platforms for the development of a wide variety of biological applications, including sensing and drug delivery (Lu et al., 2009; Saito et al., 2009; Goenka et al., 2014; Son et al., 2016). The physical, chemical and mechanical properties of carbon-based nanomaterials can be easily tuned by surface functionalization (Singh et al., 2009; Zhao and Stoddart, 2009; Lee and Geckeler, 2010; Kemp et al., 2012; Sreejith et al., 2016), allowing the modulation in their properties and functions in a remote way. On the other hand, the ability to regulate the properties of those nanomaterials by application of external stimuli is promising for several applications, that range from the development of molecular junctions, optoelectronic devices and field-effect transistors (Guo et al., 2005) to their use as sensors and drug delivery devices in the biological field (Feng et al., 2012; Zhang et al., 2016). In the last decade, photochromic molecules, such as azobenzenes (ABs) and spiropyrans (SPs), have been used for the functionalization of CNTs (Del Canto et al., 2010) and graphene (Zhang et al., 2010) for generation of optically responsive nanomaterials (Figure 1).

**Graphical Abstract:**
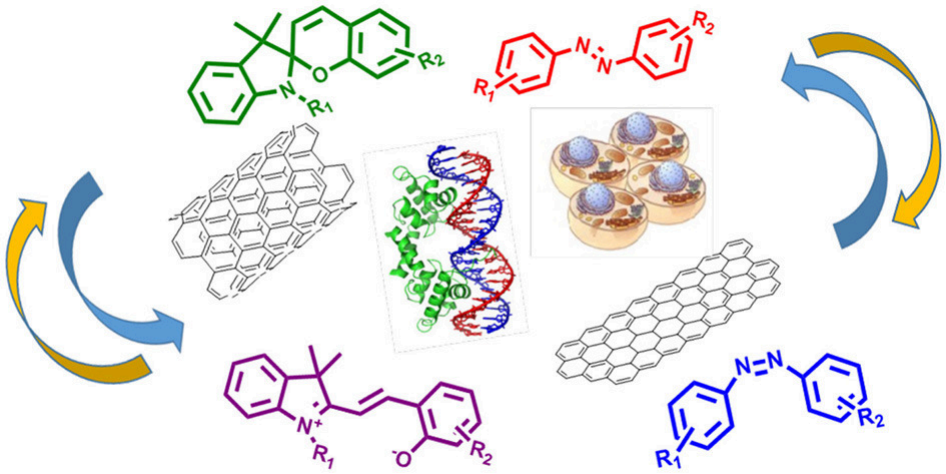
Graphene and carbon nanotubes modified with light-controlled molecular switches provide useful tools to control and trackle biological systems.

For example, the conjugation of carbon nanotubes, that present unique electronic properties, with azobenzene units has resulted (Simmons et al., 2007) in the development of photo-controlled transistors, diodes, electrodes and color detectors, where the azobenzene units act as junctions and charge transporters between two electrodes. Photo-switchable vertical junctions have been prepared from the conjugation of azobenzene units on electrode surfaces made of graphene. The resulting device presents stability both under mechanical stress and also after a large number of reversible photo-switching cycles between ABs' *trans* and *cis* isomers. An experimental and theoretical study (Zhou et al., 2009) on a nanoscale color detector based on a single-walled carbon nanotubes (SWCNTs) non-covalently functionalized with photochromic azobenzenes, has demonstrated that the change in the dipole moment of the photochromic molecule upon adsorption of photons results in the charge transfer between the molecule the material. Hybrid materials based on carbon nanomaterials and molecular switches exhibit excellent properties in the transduction of the light into electrical signals, rendering it possible to develop functional materials for solar energy storage. Recently, the Grossman group (Kucharski et al., 2014) has harnessed the combination of CNTs with azobenzenes in order to achieve energy storage hybrid materials; the proposed structure presented high turnover rate and stability. In addition, they have found that the amount of energy stored per AB molecule was redouble when the ABs were linked to the CNTs.

Currently, one of the most appealing challenges of photoresponsive materials is represented by their interface with biological systems. The efficient integration of smart materials, such as photo-responsive graphene and CNTs, with proteins, cells or tissues, is expected to pave the way for controlling the properties of the biological environments by simply applying an external stimulus. The remarkable results obtained in the past by these multi-functional nanomaterials in the fabrication of optoelectronic devices along with the continue need of novel smart materials that are able to control and tackle the functions of biological systems have made these photo-responsive nanomaterials promising tools for applications in the biological and biomedical fields. In the last decade, photo-switchable compounds (Beharry and Woolley, 2011) have been conjugated to biological macromolecules, including proteins, enzymes and DNA, to regulate their folding or to switch on and off their activity in order to control a specific enzymatic pathway or the binding preference of a protein. The resulting switchable biomacromolecules can find applications in designing drug delivery systems capable of controlling the release of pharmaceutically active compounds in time and space. Indeed, carbon based nanomaterials (Battigelli et al., 2013; Chen et al., 2013) have provided a unique scaffold for the development of nanodevices for the delivery of therapeutic agents, including anti-tumor, anti-microbial, anti-inflammatory drugs as well as for the recognition of molecules and bio macromolecules given the possibility to functionalize their surface (Zheng et al., 2003) with peptides (Lin et al., 2004), carbohydrates, antibodies and enzymes (Patolsky et al., 2004). The ability to tune the electrical as well as the optical properties of such materials and to control their surface-binding properties by suitable functionalization with proper ligands have provided much insight into the fabrication of sensitive and selective biosensors (Feigel et al., 2011; Münzer et al., 2013; Bisker et al., 2016). Possible applications of these materials could also interest the vegetal world where they could be used to regulate a large variety of functions (Giraldo et al., 2014). Great attention has been focused on the investigation of azobenzenes, thanks to their unique properties, in the biological environment as documented by recent publications (Szymanski et al., 2013). Pharmacological studies have been recently carried out on derivatives of azobenzene for the activation of biological receptors by light (Westphal et al., 2017). The combination of these classes of molecules with carbon nanomaterials have been less investigated in the biological context if compared with their applications in optoelectronic devices, solar thermal storage, memory devices or field effect transistors have been improved. Several reviews regarding photo switchable molecules and carbon based nanomaterials have been published (Feng et al., 2012; Zhang et al., 2016) in the last years; these reviews cover the preparation and the properties of these light responsive materials and their applications for the realization of optoelectronic devices and sensors. Nevertheless, only a brief space has been dedicated to discuss the properties of these nanostructures in the biological realm. In this review, we highlight the main physicochemical properties of photochromic nanomaterials derived by the combination of carbon nanomaterials, particularly carbon nanotubes and graphene, with photochromic molecules, azobenzenes and spiropyrans, focusing our attention on their interface with biological systems and the possibilities to develop nanodevices for biosensing and drug delivery applications.

## Overview of azobenzenes and spiropyrans and their biological applications

**Scheme 1:**
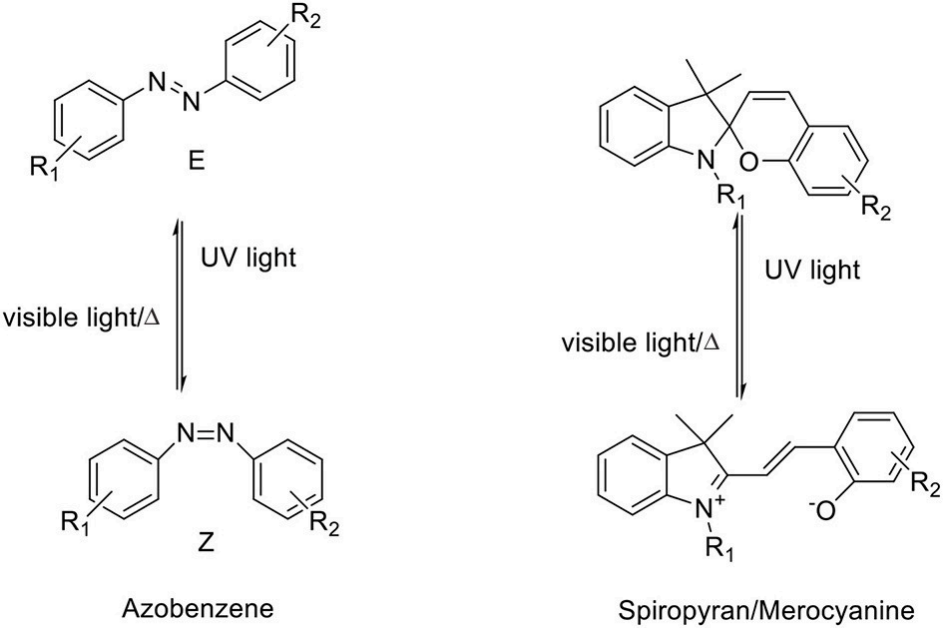
Chemical structures of azobenzene and spiropyran in both their isomeric forms.

The photo-triggered isomerization of photochromic molecules between at least two stable or metastable states by the absorption of an electromagnetic radiation can involve different types of chemical processes which allows to divide these compounds into several classes, (Natali and Giordani, 2012a) including: azobenzenes, fulgides, diarylethenes, dhyidroindolizines, chromenes, stilbenes and spiropyrans/-oxazines. The isomerization of photochromic compounds like spiropyrans/-oxazines, fulgides and diarylethenes occurs *via* peryciclic reaction while azobenzenes and stilbenes present an E/Z isomerization. Moreover, some of these compounds can also convert between their forms in response to other external stimuli including electrical input, protonation and complexation with cations or anions (Natali et al., 2010). Among the molecular switches, azobenzenes and spiropyrans have been widely studied (Raymo and Giordani, 2001; Raymo et al., 2003) and employed to fabricate responsive materials because they can be reverted to their more stable isomer either photochemically or thermally. They provide an easy access to switchable materials with a relatively ease of synthesis, high chemical stability (in both isomeric forms) and the possibility to incorporate multiple organic functional groups to allow the coupling with biomolecules as well as several types of materials, including polymers (Mosciatti et al., 2016) and nanomaterials (Weber et al., 2016) (Scheme 1, Table 1).

**Table 1:** Summary of the basic properties of azobenzenes and spiropyrans.

**Name**	**Isomerization process**	**Isomers**	**Properties**
Azobenzene	π-π*(E–Z)	E(almost planar)-more stable	Change in dipole moment
	n–π*(Z–E)	Z(bent conformation)-less stable	Significant conformational change - Low photobleaching rate
Spiropyran	Cleavage of the C-spiro-O bond and	Spiropyran (SP)-more stable	Significant change in dipole moment
	*cis-trans* isomerization of the double bond	Merocyanine (MC)-less stable	Evidence of chromatic change Presence of fluorescence only for MC isomer

Aromatic azo-compounds, better known as azobenzenes, have been discovered and used as dyes and pigments since the end of XIX century (Zollinger, 2003) while their photochromic properties and their reversible isomerization have been elucidated in the first half of 1900s (Hartley, 1937; Krollpfeiffer et al., 1938). The reversible isomerization process of AB derivatives involves the switch of the geometry of the N = N double bond that could shift between the *trans* and *cis* conformations. When the more thermodinamically stable *trans* (or E) isomer is exposed to UV light, it converts to the *cis* (or Z) metastable form (Tamai and Miyasaka, 2000), that, being the less stable, reverts spontaneously back to the *trans* form with an high quantum yield following removal of the UV source. This re-isomerization can be gained in less time by heating or by exposition of the *cis* form to VIS light. The kinetic of both isomerization reactions, i.e. *trans/cis* and *cis/trans*, are dependent on the substituents present on the molecules and their relative positions respect to the phenyl rings (Garcìa-Amoròs and Velasco, 2012). The isomerization results in a reversible variation of the following physicochemical properties: (i) the dipole moment, (ii) the spatial molecular conformation, (iii) the redox potential, (iv) the dielectric constant, (v) the fluorescence intensity, (vi) the refractive index and (vii) the absorption spectra. The nitrogen atoms of the AB molecules present a lone pair electron, and thus a n-π^*^ electronic transition is observed, in addition to the π-π^*^ transition. The first transition, at lower energy, occurs between 430 and 440 nm for both AB isomers, while the second occurs in the UV region. The latter transition is the most significant to define the isomerization process and it decreases during the *trans-cis* isomerization and switches back when the stable form is restored while the n-π^*^ electronic transition presents an opposite behavior. These bands are very sensitive to the presence of substituents on the AB backbone as well as to the solvent's effects, such as the interaction between the solvent and functional groups or bathochromic shifts due to the polarity of the solvent itself (Griffiths, 1972). Several synthetic strategies have been proposed for the preparation of the AB's photo responsive scaffolds. The most frequently used approach involves the azo coupling reactions (Merino, 2011), but also the Wallach and Mills reactions are good synthetic routes to obtain these compounds. Moreover, the azobenzene backbone can be chemically modified with a variety of molecular receptors to impart specific binding properties. For example, ABs modified with a series of crown ethers have been prepared (Shinkai and Sato, 1982), and the isomerization between the two azobenzenes' forms controls the supramolecular assembly between the molecule and the guests, i.e. metal ions. The binding between the crown ether on the azobenzene and the guest molecule results in a change of the spectroscopic properties of the azobenzene opening the way to the use of these modified ABs as supramolecular sensors (Shinkai et al., 1982). In addition, the conjugation of azobenzenes with biomolecules has allowed the exploration of these photo-switchable compounds in the biological field. The coupling of AB to biomolecules, such as peptides, allows a very precise spatio-temporal control of the biological activity of the conjugate by using light as external stimuli (Beharry and Woolley, 2011). As examples, the photo-control of the folding of β-hairpins has been achieved by incorporating (Aemissegger et al., 2005) an AB unit into an amino acid sequence that is known to fold in β-hairpins structure in aqueous solution. The light-induced switching of azobenzene from *trans* to *cis*, induced by the irradiation of UV light, results in a conformational change of the molecule, that allows to control the assembly and disassembly of the β-hairpins. Erlanger *et al* (Bartels et al., 1971) have demonstrated a precise photo-control of the activity of acethylcholine receptor by attaching azobenzene-modified choline agonists near to the allosteric sites of the receptor; the isomerization of the azobenzene allows the on-off control on the receptor itself. Other applications involving the conjugation of AB with nucleic acids, oligonucleotides, proteins and lipids (Beharry and Woolley, 2011) have demonstrated the versatility of the AB molecules as smart components for biological applications and for photo-pharmacological studies (Westphal et al., 2017).

Spiropyrans, Lukyanov and Lukyanova (2005) and Vandewyer et al. (1969) also known as spirochromenes, are photochromic compounds characterized by a twisted structure made of two heterocyclic units linked to each other through a tetrahedral carbon atom, the spiro-junction, shared between the two heterocycles; in this way, the two halves of the molecule result in two orthogonal planes. The closed stable isomer form is named spiropyran (SP) and, due to its conformation, the π-electron conjugation between the two heterocycles (indolenine and benzopyran) cannot occur in this state; the spiro C-O bond breaks and the *cis-trans* isomerization of the double bond occurs upon exposure to UV radiation. The open metastable isomer is called merocyanine (MC) and it shows a planar conformation and a delocalized π electron system (Ernsting and Arthen-Engeland, 1991). These two isomers present well known different chemical-physical properties; while the MC isomer in solution shows a strong absorption in the visible region with a maximum around 550 nm, the SP isomer absorbs intensely in the UV region usually between 200 and 300 nm. Moreover, MC isomers show fluorescence emission in the visible range (Barachevsky, 2000). The switching process is reversible and the MC conversion to the stable SP form is restored with exposure to visible light or heating the sample in dark conditions (Görner, 2001; Minkin, 2004). Furthermore merocyanine can be stabilized through coordination with metal ions (Natali and Giordani, 2012b) allowing the SP-MC isomerization to occur also in the dark (Zakharova et al., 2009; Baldrighi et al., 2016). The improvement of the complexation of spiropyrans with metal cations by chemical modifications of the substituents on the spiropyran moiety (Chibisov and Görner, 1998; Sakata et al., 2008) has allowed the development of this class of photochromic molecules for sensing (Winkler et al., 1998). Two main approaches can be followed for the synthesis of spiropyrans: the condensation of an indolenine base bringing an active methylene group with an *o*-hydroxy aromatic aldehyde (Wizinger and Wenning, 1940) or the condensation of *o*-hydroxy aromatic aldehyde with the salts of heterocyclic cations (Lukyanov and Lukyanova, 2005).

**Figure 2:**
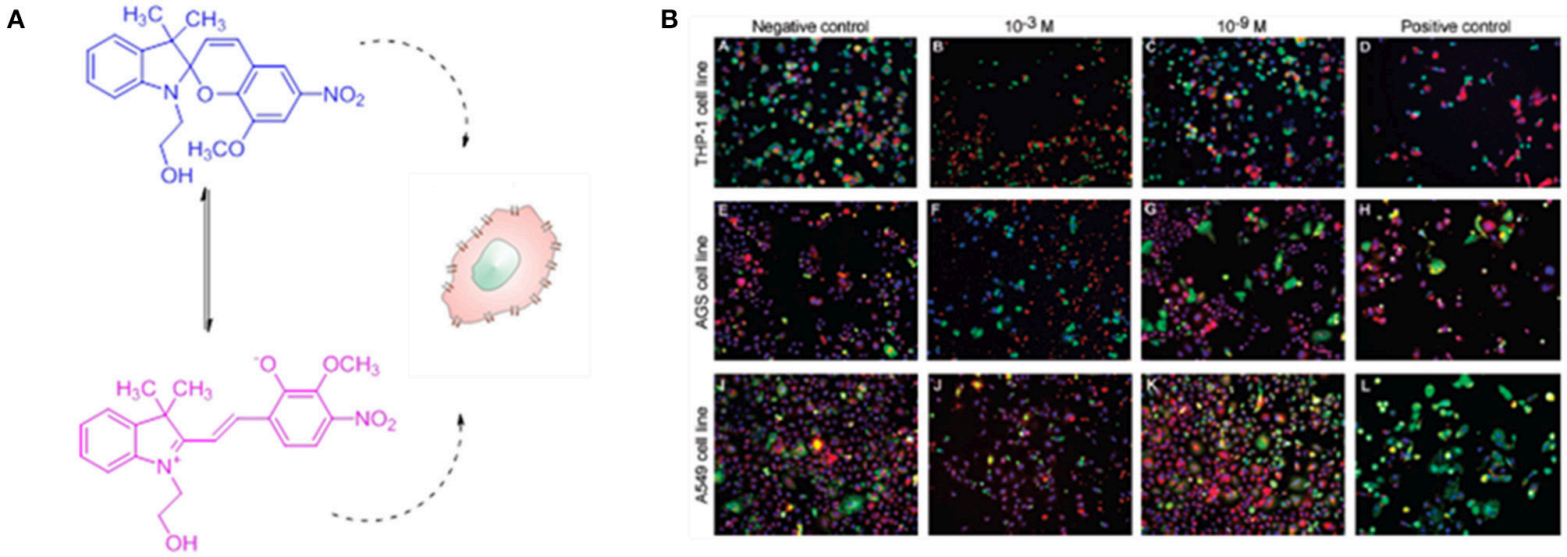
*In vitro* cytotoxicity of two forms of spiropyran. **(A)** Chemical formula of the 8-methoxy-6-nitro-BIPS used in the toxicity study in the two different isomeric forms. **(B)** Confocal images of three cell lines: human monocytic leukemia cell line (THP-1), human gastric cancer cell line (AGS), and human alveolar epithelial cell line (A549), treated with SP solutions at concentrations of 10^−3^ M and 10^−9^ M after 24 h of exposure. The negative and positive controls are also shown. The images report the cell stained for (i) nuclei (blue), (ii) cell membrane permeability (green), (iii) lysosomal mass/pH changes (red). A significant decreased cell viability and an increased membrane permeability has been detected at concentration of 10^−3^ M, while the cell viability is comparable to the negative controls at concentration of 10^−9^ M. Reprinted with permission from Movia et al. (2010a). © 2010 American Chemical Society.

The toxicological properties of spiropyrans and azobenzenes derivatives have been investigated for their application as biological light controlled nanosensors, drug delivery nanovehicles and nanofluidic systems. The *in vitro* toxicity response of SP bearing methoxy and nitro moieties on the backbone has been investigated (Movia et al., 2010a) in three different cellular lines i.e., human monocytic leukemia cell line (THP-1), human gastric cancer cell line (AGS), and human alveolar epithelial cell line (A549) (Figure 2).

**Figure 3:**
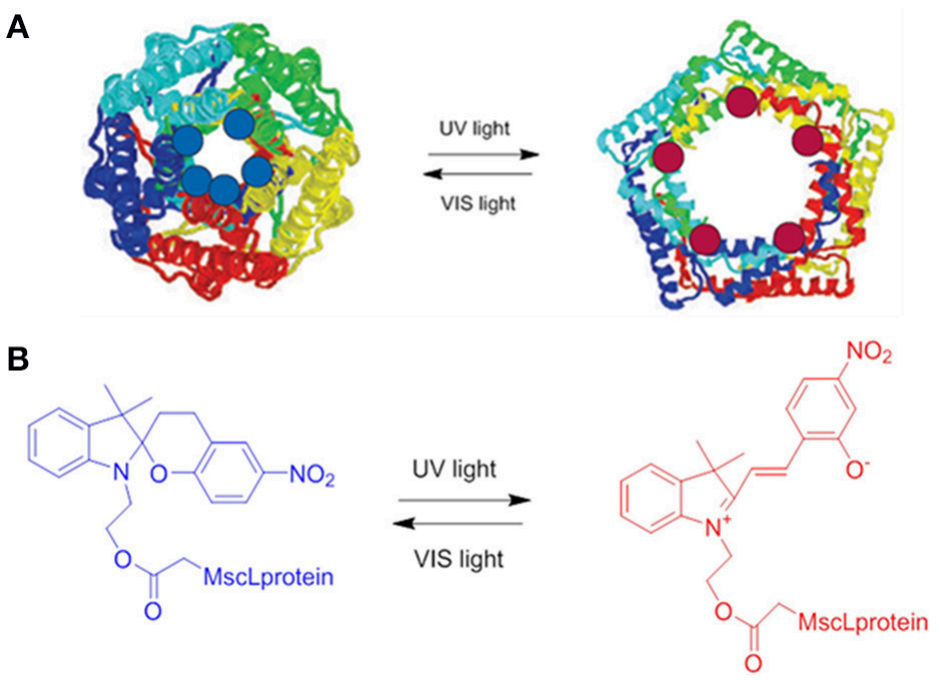
**(A)** Schematic representation of the MscL protein functionalized with SP; the switching from the SP (blue dots) to the MC form (red dots) upon irradiation with an UV source at 366 nm results in the opening of the protein. **(B)** Chemical structure of SPs used to functionalize the channel protein in its two isomeric SP and MC forms (Kocer et al., 2005).

This investigation has shown that toxic effects on the cell lines tested are very unlikely at concentration between 10^−4^ and 10^−9^ M for 24 h in cell media at 37°C, however a minimal toxicity appeared after 72 h of continuous exposure at concentrations of 10^−3^ M (Figure 2B). On the other hand, studies on the degradation process of the molecule has evidenced how the hydrolysis of the compound occurs already after 24 h of exposure, which could represent a limitation for the *in vivo* applications of these compounds. Several studies have focused on the applications of SP entities in biological environments, as examples the interaction between SP and MC forms with a large variety of proteins (Amdursky et al., 2016), from human serum albumin (HSA) and insulin to lysozyme, has been investigated. These studies have evidenced that the optical properties of the photochromic entity are maintained upon its interaction with the protein but they are influenced by the presence of the proteins itself. Indeed, the non-covalent interactions between the protein and the molecules improve the solubility of the photochrome, decreasing its aggregation in aqueous solution, while it stabilizes the isomerization to the closed SP form. On the other hand, the presence of SP units have shown a clear influence on HAS protein, as evidenced by the increase (5-fold) of the electrical conductance through the protein itself. By coupling a SP derivatives to the residue Cys-374 in G-actin enzyme, Sakata and colleagues (Sakata et al., 2005) have found a way to inhibit the interaction between the enzyme and its binding proteins by switching the SP unit with light. The group of Feringa has reported (Kocer et al., 2005) the light-controlled activation of a channel protein from *E. Coli*, the mechanosensitive channel of Large conductance (MscL), by coupling the channel itself with a synthesized cysteine-selective iodoacetate moiety attached with a SP unit (Figure 3). Under irradiation with UV at 366 nm the isomerization of the SP unit results in the reversible opening/closing of the molecular valve.

The encapsulation of the functionalized protein within the liposomes has allowed the external photochemical control over transport through the channel, opening the way to study these conjugated systems in more complexes biological environments. The preparation of polymeric materials which respond to light showing reversible photochromism has been achieved by incorporating SP (Kundu et al., 2014; Ventura et al., 2014) or AB (Kundu and Pintu, 2014) photo-responsive units in nanoporous polymeric frameworks. The functionalization of the polymer backbones with the photochromic molecules results in the possibility to modulate different properties of the polymer itself, including the polymer solubility, volume phase transition, mechanical properties and to control the capture and release of metal ions maintaining the properties of the nanoporous polymeric structure (Klajn, 2014).

The properties of photo-switchable molecules discussed herein have been exploited and enhanced by conjugation with carbon nanomaterials. The crucial point in this context has been represented by the significant different properties, such as geometries, electronic properties, physical and chemical features, of the isomers of AB and SP, making these two molecular switches promising components for the preparation of photo-switchable carbon nanomaterials.

## Carbon nanomaterials conjugated with azobenzenes and spiropyrans: preparation and properties

**Scheme 2:**
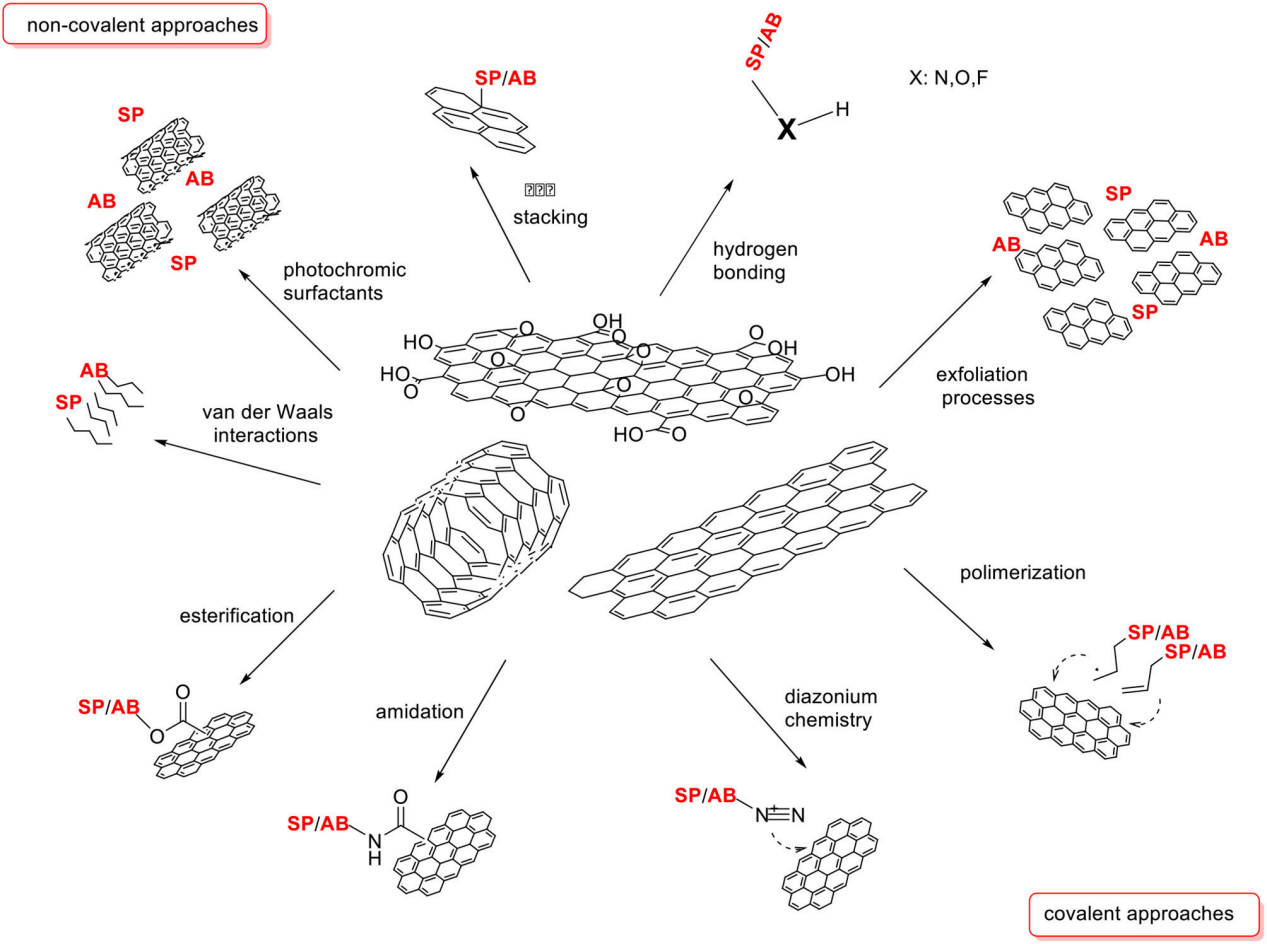
Non-covalent and covalent approaches for carbon nanomaterials functionalization.

Carbon nanomaterials are versatile platforms in nanobiotechnology thanks to their chemical stability, biological compatibility and large surface area as well as the possibility to chemically modify their surfaces, both covalently and non-covalently (Scheme 2), in order to tune their physicochemical properties.

We focus our attention on the functionalization of carbon nanomaterials with photochromic molecules in order to obtain optically responsive CNTs and graphene, that represent the main carbon nanomaterials studied in the biological field although also examples of fullerenes functionalized with SPs (Xu et al., 2002) and ABs (Kay et al., 2002) have been reported for other applications. Among carbon nanomaterials, carbon dots have been deeply studied with good results for biological purposes due to their photochemical properties and their bicompatibilty (Sun et al., 2006; Zheng et al., 2015) although their photochemical properties are not due to the conjugation with molecular switches.

Since their discovery in 1991 (Iijima, 1991), CNTs have been identified as promising scaffold in nanotechnologies, thanks to their remarkable mechanical and physicochemical properties (Popov, 2004), and they have been applied in microelectronics, including memory devices and supercapacitors (Fan et al., 2010), and in the biomedical area as drug-delivery devices (Battigelli et al., 2013) and biosensors (Arias De Fuentes et al., 2011; Kruss et al., 2013). Single-walled carbon nanotubes (SWCNTs), rolled-up cylinders of graphene sheets, present a diameter of 0.4–2 nm and lengths up to several cm. Due to their interesting properties, SWCNTs along with the multi-walled carbon nanotubes (MWCNTs), which are formed by multiple cylinders stacked in each other, have been deeply investigated in term of toxicity and biocompatibility *in vitro*, in an ample variety of cell models (including neuronal cells and osteoblasts; Smart et al., 2006), and *in vivo* models including intravenous administration tests with functionalized MWCNTs in mice (Lacerda et al., 2008). Graphene-based materials have emerged over the last decade as scaffolds in the context of many applications, including biosensing and cell imaging. Graphene, single-atom-thick sheet of carbon atoms arrayed in a honeycomb pattern, presents unique mechanical, electronic and optical properties. Because of such properties as well as the possibility to modify its surface with a variety of functional groups, graphene and graphene oxide (GO) are considered promising materials for biological applications. The results obtained from biological investigations using carbon nanomaterials (Bianco et al., 2011; Bianco, 2013) focus the attention on the importance of the purification procedures of these materials in order to have safe and biocompatible materials. Indeed, CNTs or GO with graphitic residuals have shown toxicity issues.

The surface functionalization of CNTs and graphene is fundamental in order to improve the stability and dispersion in water, particularly useful for their applications in the biological field. For example, the as-prepared SWNTs aggregate into bundles due to strong van der Waals interactions, a condition that strongly affects their physical properties and reactivity. Therefore, surface functionalization has tremendous implication on the stability of CNT dispersion. Stable aqueous dispersion of CNTs can be obtained by means of physisorption of a series of surfactants. For example, anionic surfactants allow an efficient dispersion of SWCNT in high concentrations in aqueous solution resulting in a highly charged tubes with a value of zeta potential, an index used to evaluate the magnitude of the electrostatic interaction between colloidal particles, of roughly −95 mV (White et al., 2007). These results constitute a clear evidence of how these materials could be functionalized in non-destructive ways to maintain the properties of the material itself, allowing their further surface functionalization (Singh et al., 2009; Karousis et al., 2010). Moreover, these functionalization approaches allow the introduction of new functions that can be used to tune the final properties of the hybrid system. This is the main goal of the hybrid photochromic architectures. Non-covalent functionalization usually occurs by physical adsorption of modified ABs or SPs on the carbon nanomaterial using π-π stacking and electrostatic interactions. By using the latter strategy, the sp^2^ structure of the nanomaterial is only moderately perturbed. In addition, ABs and SPs can be used in a non-covalent approach for a molecular-assisted liquid-phase exfoliation (Döbbelin et al., 2016). In this study the ability of alkoxy-substituted azobenzenes, which act as surfactant, to increase the yield of graphene exfoliation in NMP has been tested either in dark or UV irradiation conditions. The obtained results have shown an increase of the exfoliation yield up to 80% compared to the solvent alone and the exfoliation rate in terms of number of layers and defects of the final material have remained almost around the same values. Vijayakumar et al. (2011) have reported the synthesis of AB polymers containing pyrene functionalities that have been used for the non-covalent functionalization of CNTs, which occurs by the conjugation of pyrene and CNT via π-π interactions. The resulting hybrid material has shown an increased solubility in several polar and apolar solvents and good thermal stability; solubility values between 0.2 and 0.4 mg/mL in THF have been noted and good polidispersivity index values have been recorded for such systems. In addition, a faster kinetics of photo-isomerization has been observed for the ABs' moieties on the nanomaterial surface in comparison to the polymer alone. Using the same π-π stacking approach, SPs functionalized with pyrene units has been employed for the non-covalent functionalization of SWCNTs (Setaro et al., 2012), obtaining an hybrid material with good light-responsive properties due to the preservation of the SPs switching capabilities on the CNT surface. Similar π-π interactions were exploited to functionalize graphene/graphene oxide sheets (Song et al., 2014) and carbon nanotubes (Perry et al., 2015) by using perylenediimide or pyrene modified SPs resulting in a photo-switchable carbon based materials. The binding affinity of SP toward divalent cations, particularly Zn^2+^ is retained on all the surface of the carbon nanomaterials and the prepared hybrid materials exhibited SP-MC isomerization when are exposed to Zn^2+^, representing an example of surface confined SP receptor. Non-covalent electrostatic interactions between azobenzene-based surfactants with aliphatic chains and glycerol-dendrons have been exploited for the reversible solubilization of CNTs. The critical micelle concentration of the hybrid system varies significantly between the *trans* and *cis* isomer. The photo isomerization from the *trans* isomer to the *cis* one increases the critical micelle concentration up to 11 times (Kördel et al., 2012). The possibility to switch the conformation of the AB entity exposing the surfactants to UV light allows a variation of the supramolecular conformation of the surfactants' micelles and consequently a different rate of dispersion of CNTs; the light-controlled supramolecular CNTs assemblies prepared in this way are suitable for several applications. A similar approach has been used to assembly graphene sheets regulated by controlling, upon irradiation with UV light, the polarity of an azobenzene derivative (Chen et al., 2015). The zeta potential of the hybrid system increases with the molar weight of the azobenzene derivative. At concentrations of AB below 0.6 mM, the zeta potential rapidly switched from a negative to a positive value, showing that the AB units have been attached to the surface of the material and have changed the charge density. When molar weight equals 0.6 mM, the zeta potential becomes stable implying that the surface of the GO is fully modified by the switchable molecules. The modification of CNTs by using SP moieties *via* van der Waals interactions has been investigated using microscopic techniques. These studies have focused on the orientation of the dipole moment in relation to the CNTs' axes, its density and the distribution of the molecules on the surface (Malic et al., 2011). Spectroscopic investigation demonstrated a redshift of the transition energy upon isomerization of the SP, demonstrating how these hybrid materials are suitable for the optical readout of the molecular switches.

Covalent functionalization of the nanomaterial with the photochromic entity allows robust modification and a precise control of the functionalization's degree. The common approach for the covalent functionalization consists in the oxidation of the carbon materials following the attachment of modified AB or SP by condensation reactions. Graphene oxide presents carboxylic groups and other functional groups on the surface (Dreyer et al., 2010; Kemp et al., 2012) that can be readily used for covalent modifications. Condensation reactions, such as esterification and amidation reactions, are the preferred approaches: the carboxylic groups on the surface of the nanomaterials are activated by generating the corresponding acid chloride that reacts with the suitable SP (Khairutdinov et al., 2004) or AB (Feng et al., 2007) moieties carrying OH or NH_2_ reactive groups. Diazonium reactions involving the desired photochromic compounds have been performed directly on pristine CNTs: (Sadowska et al., 2009; Wang et al., 2009) ABs, for example, have been covalently linked using isoamyl nitrite in polar solvents under mild conditions. The covalent functionalization of CNT has also been achieved by radical polymerization of azobenzenes' monomers bearing a methacrylate functionality that reacts with surfaces exposing bromine groups of modified CNTs in presence of CuBr/HMTETA (1,1,4,7,10,10-hexamethyltriethylenetetramine) as catalyst, yielding to the surface functionalization of CNTs (Hu et al., 2011). Other polymerization approaches involving azobenzene monomers have been employed to covalently functionalize the surface of graphene: single layered graphene, obtained from reduction of GO, has been functionalized in order to have a graphene based initiator which has been subsequently polymerized *via* diazonium chemistry with the appropriate azobenzene derivative (Wang et al., 2011). The light responsive features of photochromic compounds with their peculiar properties, which can be tuned with a variety of functional groups, and the different carbon nanomaterials allow the realization of multiscale responsive materials controlled both in space and time. Azobenzenes and spiropyrans present deep differences in the modulation of the dipole moment, charge transfer and current change due to their different chemical scaffold. The variation of dipole moment in azobenzenes is strictly due to the *trans-cis* photo-isomerization that involve the azo moiety, a process that imply the change in the geometry as well as in the symmetry of the compound. These changes are translated in a variation of the distance between the charges present in the molecule, closest for the *cis* isomer, resulting in the alteration of the dipole moment. The peryciclic isomerization that involves the stable SP form of spiropyrans and conducts to the MC form of the compound implies a significant charge separation resulting in an increased value of dipole moment from ~4–6 D, for the SP form, to ~14–18 D, for the ring opened MC isomer (Klajn, 2014). The possibility to synthesize ABs and SPs molecules with different functional groups has allowed to exploit different properties (i.e., solubility, polarity, reactivity etc.) within the same class of compound. This change in the properties affects the final hybrid materials in different ways, particularly when attached on the nanomaterial surface. Indeed, the degree of functionalization and the covalent or non-covalent modification of the surface of the material deeply influence the final properties. The interactions between molecules and materials, the different properties of the carbon materials used (CNTs or different type of graphene i.e., GO, rGO, graphene monolayer, graphene ink) and, last but not least, the experimental sets used for the test of the materials in the different applications fields (i.e., the time of irradiation exposure and the precise wavelength used, the environmental conditions of solvent and temperature, the level of vacuum or the presence of inert atmosphere etc.) have opened the way to tune the properties of these materials in a precise and desired way. The most significant properties of these hybrid-material will be discussed with application focused in the biological field. The chemical functionalization of the nanomaterial with ABs or SPs implies that, when the isomerization reaction occurs, a variation of the dipole moment appears and as a consequence this property influence also the carbon nanomaterial's scaffold. Theoretical investigations (Simmons et al., 2007) on azobenzene-CNT conjugated system have demonstrated a large orbital overlap between the phenyl moieties of the azobenzene unit in the *trans* stable conformation and the CNT surface. This effect, in addition to the presence of an electronegative NO_2_ group on the molecule, gives to the compound a significant dipole moment of ~9 D. When the molecule is exposed to UV light the isomerization of the AB unit to the *cis* conformation results in a reduced orbital overlap that give a smaller dipole moment of ~6 D. The system returns to the original *trans* stable conformation when the UV light is removed. This theoretical study was confirmed experimentally by measuring the drain current upon irradiation of the materials with light at 254 and 365 nm. A single nanotube transistor was prepared by chemical vapor deposition techniques following non-covalent functionalization with ABs moiety. The obtained device is tested with several electrical measurements of the drain current and the related electric conductance and gate voltage in the desired condition of light exposure: the value of the shift of the gate voltage correspond to the expected calculated values.

Raman spectroscopy is usually employed to characterize the functionalization of graphene and other carbon materials (Ferrari and Basko, 2013). This technique is very suitable for carbon based materials because it is a non-destructive method presenting high resolution and allow to give precise electronic and structural information of the material. Raman spectroscopy performed on graphene functionalized AB shows that isomerization of AB to the *cis* form, corresponding to a decrease of the dipole moment, alters the extent of doping and the charge carrier concentration of graphene as observed from the downshift of the G band of around 3 cm^−1^ as well as for the position of the 2D band. Electrical measurements on these hybrid materials (Kim et al., 2012) have demonstrated that the switching of these molecules can induce variations in terms of electrical conductivity and charge transfer of the material; the UV irradiation decrease the conductivity of the material as consequence of a downshift of the Dirac point due to a further n-doping when the material is exposed to UV sources (Jang et al., 2012). In addition, the charge separated MC state of the molecule, represents a scattering site that is responsible of a light decrement of the electron mobility of the material itself. The switching events are reversible and they can be repeated for several cycles: (Kim et al., 2012) the range time in which the two isomers convert is a key aspect for the scattering properties. The functionalization of graphene with AB derivatives, enables the reversible modification of the electrical and quantum properties of the Dirac fermions by switching the AB between the *cis* and *trans* conformation (Margapoti et al., 2014). The molecule in *cis* conformation allows a gating between the nearby graphene layers resulting in resonance oscillation of the current density, while in the *trans* conformation the geometry of the doped molecules doesn't allow a close contact with the substrate, the interaction is minimum and the formation of quasibound states isn't promoted. Similar results are obtained on reduced GO *via* non-covalent functionalization with SP moieties (Joo et al., 2012). Another interesting property of AB and SP-based hybrid systems is the possibility to control the induced fluorescence of carbon nanomaterials, which is very promising for applications in the field of bioimaging (Liao et al., 2013, 2015). In the reported example, the functionalization of carbon nanoparticles with SP has shifted the fluorescence of the particles from 510 nm to 650 nm at an excitation wavelength of 420 nm. The fluorescence intensity at 650 nm is increased when the hybrid system is exposed to UV light due to the formation of the MC isomer of the molecule, moreover differences in the absorption features of the functionalized material are found. In addition, the absorption typical of the closed-ring SP forms on the material is red-shifted from 340 to 355 nm when the material is exposed to UV source and a clear new absorption peak at 550 nm appears; these evidences provide an easy access to systems that can be controlled in a precise way using specific wavelengths opening the way to multimodal imaging of biological systems. SWCNTs functionalized with photochromic ABs or SPs represent a good platform for the bioimaging applications; the switching between the two isomers can be used to regulate the intrinsic near-IR fluorescence of the modified SWCNTs (Movia et al., 2010b) in biological systems.

## Biological applications of photochromic CNTs and graphene

**Figure 4:**
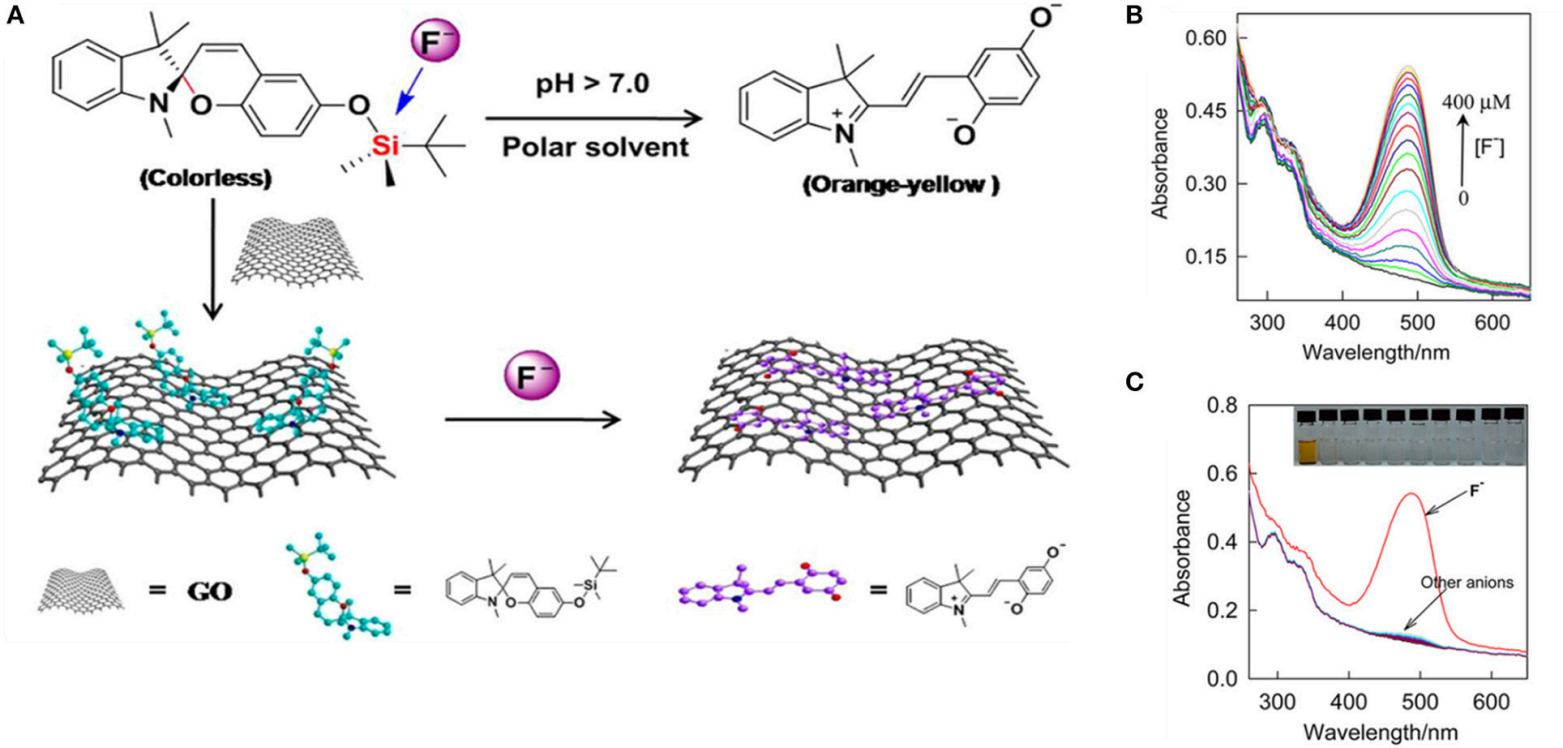
**(A)** Schematic representation of the preparation of the hybrid system SP/GO and its conversion from the SP to the MC isomer upon binding the fluoride ion, in solution upon binding on the GO surface. **(B)** UV-vis absorption spectra of the hybrid material in presence of increasing concentration of F^−^ in solution: it's evident how the increasing F^−^ concentration increase the formation of MC isomer. **(C)** UV-vis absorption spectra of the hybrid material in presence of F^−^ ions compared with other ions. The system and the isomerization of the SP is highly selective for F^−^. Reprinted with permission from Li et al. (2013). © 2013 American Chemical Society.

Several studies have been conducted for different purposes, underlining the potentials of these materials in the context of bioimaging and biosensors. For example, biocompatible graphene oxide functionalized with spiropyrans or other molecular switches (Li et al., 2013; Wang et al., 2014) have been studied for sensing of fluoride ions in cells. The fluoride (F^−^) ion is an essential micronutrient for the body growth, however it can cause neurodegenerative diseases at high level, therefore its rapid detection in biological fluids is particularly important. Li et al. (2013) have prepared an hybrid material functionalized with SP containing a silylate group that can detect fluoride (F^−^) increasing the GO's capability to adsorb liphophilic compounds on its surface (Figure 4). The conversion of the SP isomer into the MC one upon binding of fluoride ions allows their colorimetric quantification; a detection limit of 9 × 10^−7^ M in aqueous media has been achieved by this system. Preliminary studies on bovine serum evidenced the effective uptake of the anion by the SP unit, which is enhanced when this moiety is coupled to GO probably due to the GO capability to adsorb liphophilic compounds on its surface. The system is pH dependent and the selective detection of F^−^ ions in respect to others, such as Cl^−^, Br^−^, I^−^, ${\text{HSO}}_{4}^{-}$, ${\text{NO}}_{3}^{-}$, ${\text{ClO}}_{4}^{-}$, AcO^−^, ${\text{HCO}}_{3}^{-}$, and H_2_${\text{PO}}_{4}^{-}$, opens the possibility to use this material for bioanalytical applications.

**Figure 5:**
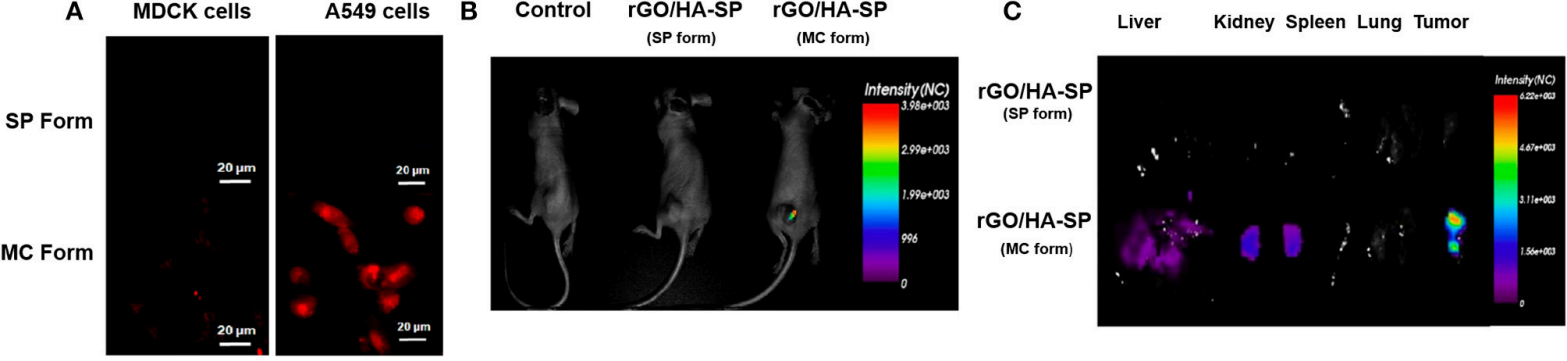
**(A)** Confocal images of rGO/HA-SP in MDCK and A549 cells after 2 h of incubation, showing the fluorescent properties of MC isomers and their use as fluorescent probes. **(B)** Bright fluorescence images obtained from the tumors of mices treated with rGO/HA-SP in both isomeric states. **(C)** *Ex-vivo* images of several organs highlighting the targeted delivery of the hybrid systems. Liver and kidneys show strong fluorescence. The accumulation in liver could be due to the effect of circulating blood and to the uptake of the nanosized rGO/HA-SP material by the SRE system. The high fluorescence in kidneys indicate a rapid excretion of the composite material. Reprinted with permission from Al Nahain et al. (2013). © 2013 American Chemical Society.

A novel electrochemical sensor for the detection of F^−^ ions in biological fluids has been recently developed from the non-covalent assembling of a SP moiety on SWCNTs-modified glassy carbon electrode. This hybrid material has been shown to be able to selectively detect F^−^ ions in the nano molar range (Tao et al., 2016). The potentiality of photochromism and thermochromism systems for sensing a variety of biological relevant molecules is in rapid growth for several applications in the biological context (Avella-Oliver et al., 2016). Fluorescent nanoparticles of reduced graphene oxide, functionalized with hyaluronic acid (HA), as targeting subunit, and SPs, as fluorescent probes, have been prepared for cellular imaging and drug delivery applications (Al Nahain et al., 2013). First of all, the spectroscopic properties of these hybrid systems have been monitored by UV-vis and fluorescence spectroscopies, thanks to the fluorescent properties of the SP unit. A strong fluorescence signal is obtained from the spiropyran unit after irradiation with UV light at 365 nm upon conversion to the MC isomer. Investigations on cancer cells by using confocal microscopy have highlighted an efficient delivery of doxorubicin in cancer cells over normal ones due to the presence of the HA targeting subunit (Figure 5). In addition, *in vivo* studies on mice model have shown the aggregation of this hybrid material loaded with doxorubicin in particular in tumor tissues, as revealed from the emission of the MC isomer. The low toxicity of the multifunctional nanomaterials has been evidenced both *in vitro* and *in vivo* investigations, demonstrating how these systems hold promises as fluorescent probes and anticancer drug carriers.

**Figure 6:**
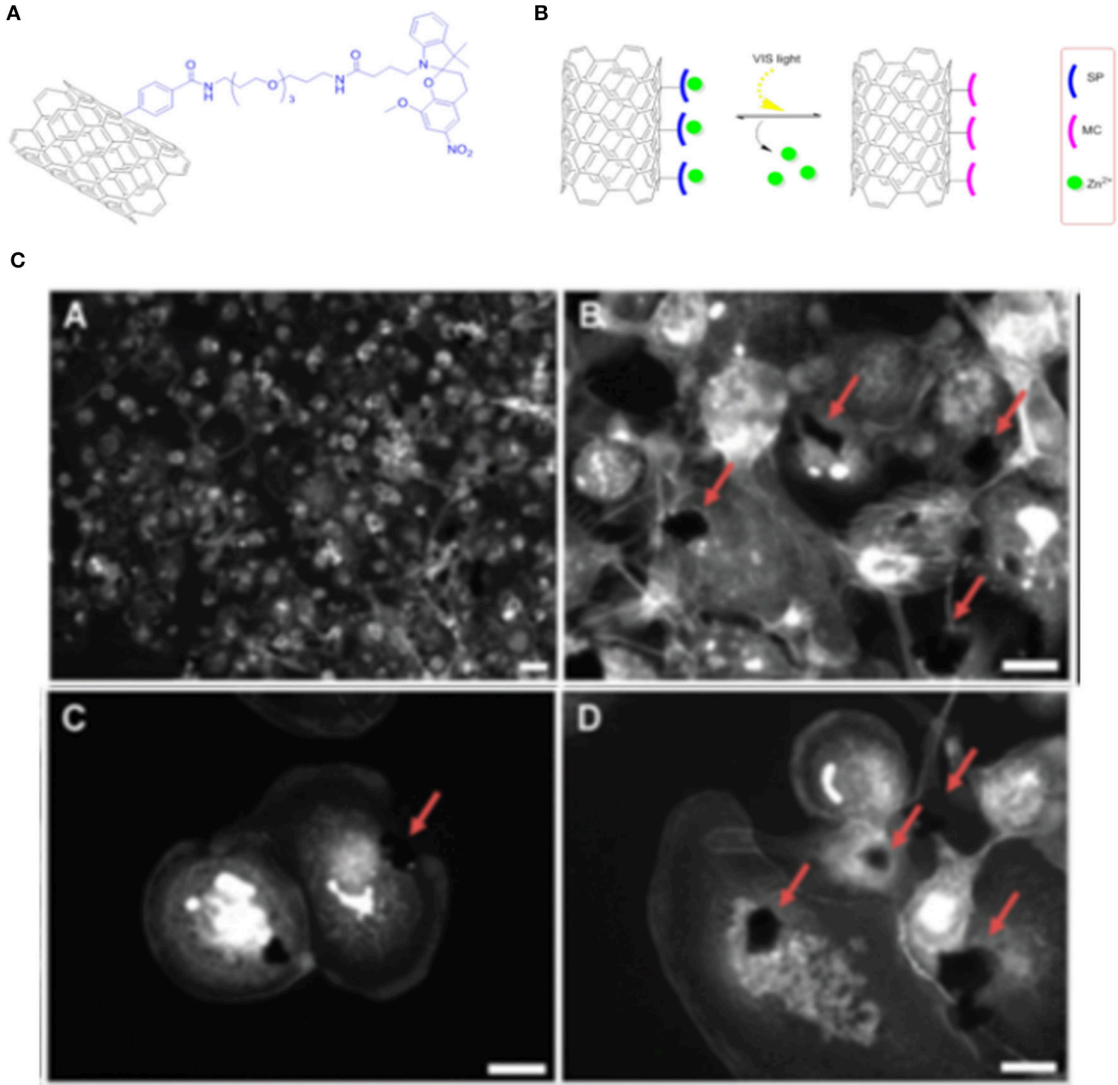
**(A)** Structural formula of the sensing system for Zn^2+^ obtained with covalent functionalization of oxidized SWCNTs with the SP. **(B)** Schematic representation of photo-switchable release of Zn^2+^ with hybrid SWCNTs-SP systems. **(C)** Epifluorescent microscopy images of PMA-activated human monocytic leukemia cells treated for 24 h with the described SWCNTs underline how “macrophage-like” cells grow in contact with the nanotube aggregates. Reproduced with permission from Del Canto et al. (2012) with permission of the PCCP Owner Societies.

Moreover, SPs can be mixed with other fluorophores (Flavin et al., 2012) for the preparation of multifunctional materials, such as photo and pH tunable materials. SWCNTs modified with SP and azadipyrromethene moieties have been prepared for bioimaging applications. The development of systems by combining different photochromes allows (i) the application of different wavelengths and (ii) the modulation of the properties of the different units in the system by applying the proper stimuli; the obtained materials present different tunable colors, superior photo-stability and brightness for bioimaging applications. GO coupled with spiropyran-BODIPY conjugated multifluorescent polymers (Sharker et al., 2014) have been prepared for bioimaging. This photoresponsive nanomaterial, obtained by reducing GO to rGO and conjugating it with 8:2 ratio (in weight) of BODIPY to SP conjugated polymers, presents good biocompatibility. The conjugated system has evidenced stimulus-responsive imaging properties both *in vitro* and *in vivo*, with the SP backbones presenting well defined photosensitive features and the BODIPY moieties conferring pH dependent properties to the hybrid material. This fluorescence intensity of the probe increase when the pH of the environment decrease due to the presence of BODIPY moieties. Moreover, the presence of SP polymers make this material sensible to UV light, which allow the isomerization of the SP units to the fluorescent MC form. The results obtained so far in the delivery of therapeutic agents (Wu et al., 2009; Vuković et al., 2010; Benincasa et al., 2011) with carbon based materials are very promising and require deeper investigations. Photo-controllable SWCNTs have been proposed as promising scaffolds for the controlled release of zinc (II) cation, indeed preliminary investigations have evidenced the possibility to use spiropyran-functionalized SWCNT for a light-controlled release of anti-inflammatory agent Zn^2+^ (Del Canto et al., 2012; Figures 6A,B).

Functionalization of oxidized SWCNTs has been performed via the well-known “Tour reaction” (Bahr and Tour, 2001) to allow a consecutive amide coupling with PEG chains, in order to improve the biocompatibility of SWCNTs, which have been then covalently functionalized with SP moieties to give the final hybrid material. The efficient switching of the SP units on SWCNTs has been confirmed using absorption and emission spectroscopies that evidence the photochromic on-off switching cyclic properties. The uptake and release of Zn^2+^ has also been investigated by using emission spectroscopy (Figure 6C), due to the fact that the presence of Zn^2+^ allows the conversion to the MC isomer that present bright fluorescence. This hybrid material has shown excellent performance on light-activated release of Zn^2+^ in PMA-activated human monocyitic leukemia cells. These studies could open the way to more complex drug delivery systems for the light-responsive release of drugs containing metal cations in their structure or that could coordinate with a specific cation interacting with the proposed photochromic SP entity. A series of photo-switchable Zn^2+^ sensitive materials have been recently prepared by non-covalent functionalization of several types of carbon nanomaterials, including graphene, graphene oxide, carbon nanotubes, with a pyrene-appended SP as Zn^2+^ binding unit (Perry et al., 2015). The high selectivity and sensitivity obtained by these systems for binding Zn^2+^ over a range of potentially competitive cations allows its detection in complex biological media. Tao and colleagues (Tao et al., 2014) have developed an electrochemical sensor coupling a SP-β galactosidase probe to a SWCNTs unit. This system undergoes reversibly structural isomerization to the MC form of the SP units under UV light irradiation when the glycosidic bond being cleaved by the β-galactosidase, this phenomenon produces reversible redox current peaks that are enhanced by the conjugation with the SWCNTs unit. Several studies have underlined the high potentialities of this probe as an ideal candidate to determine different concentration of β-galactosidase digestion kinetics. Song and collaborators (Song et al., 2011) have prepared SP modified MWCNTs (multi-walled carbon nanotubes) to modulate the horseradish peroxidase (HRP) activity by light irradiation. Irradiation with UV light converts the SP to the MC isomer, which is responsible of the enhancement of the catalytic activity of the peroxidase. The mechanism for the modulation of the enzyme activity is not clear, however, the authors have hypothesized that the enzyme activity is controlled by the MC form that interact with the active site of the peroxidase or influence the enzyme tertiary structure assembly. In alternative, the process could be ascribed to MWCNTs high surface to volume ratio presenting high affinity for hydrophobic molecule increasing the concentration of the substrate in the active site. To find a suitable application for these SP-MWCNTs, the same scaffolds have been assembled with a particular DNA aptamer, which can bind to lysozyme, with high selectivity and sensitivity. The DNA aptamer competes with the interactions between HRP and the SP-MWCNTs. They have developed a colorimetric competitive assay for lysozyme, over produced in several diseases, with good detection limits. Such systems can be easily translated to control the activity of other natural proteins by using suitable binding ligands. Recent investigations have highlighted the ability of hybrid SP-graphene-polymers conjugated systems to control and regulate the activity of different enzymes (Parlak et al., 2016a). In these studies, the remote regulation of enzyme activity is attained in a delimited space with high spatial and temporal precision through noninvasive processes. Pyrroloquinoline-quinone dependent glucose dehydrognease was tested. A biointerface has been prepared by immobilizing the enzyme on a graphene-polyacrylamide SP methacrylate scaffold assembled on an electrode surface. Upon irradiation of the biointerface with UV light, the conversion of the SP unit to MC isomer resulted in the change of the wettability of the interface, the volume and the polarity of the polymer film along with the related electrical conductivity of the system. When the photochromic units in the polymer are in the SP form, the polymer is densely packed at the electrode interface, resulting in a low permeability of the polymer for the substrate: a low bio-electro catalytic activity has been registered. When the molecules are in the MC form, the volume of the polymer increase, the electric dipole moment difference is higher and, as a consequence, the catalytic site of the enzyme is more accessible. A more complex electrode system has been developed with two compartmentalized subunits: one built based on the previous mentioned light-responsive system and the other containing a temperature-responsive subunit showing amine-terminated poly(N-isopropylacrylamide) assembled with cholesterol oxidase (Parlak et al., 2016b). In this second compartment the reversible formation of hydrogen bonds between graphene and the polymer is regulated by the temperature: at high temperature the hydrogen bonding interactions are weaker and the natural substrate can rapidly reach the catalytic site of the enzyme. These systems are promising examples for the generation of programmable bio-electrodes, by using molecular switches and carbon based materials, with logic gates ability to control several enzymatic functions. Among biomedical applications cancer therapy is a deeply investigated research field due to the everyday demanding necessity of new therapies for tumor diseases. In this context CNTs have been deeply studied and tested in several types of cancers (Ji et al., 2010; Son et al., 2016). They have been studied as carriers for several kind of pharmaceutically active compounds, including topoisomerase I and II inhibitors, antimicrotubule agents or gene therapy factors among others. CNTs have also been tested in a wide variety of carcinomas from kidney tumor to lung and melanoma cancers. These materials have evidenced also potentialities in photodynamic cancer therapies. The contribution of photo switchable molecules in this context represents an important challenge for the biological application.

Carbon nanomaterials have found also application in the vegetal context due to their high stability and interesting chemical and physical traits (Giraldo et al., 2014) that have allowed the study also *in vivo* of photocatalytic complexes based on SWCNTs and chloroplasts. As examples, carbon nanotubes can allow electron transfer processes between themselves and the photosynthetic machinery increasing the light energy capture in a broad range of wavelengths in UV, visible and NIR regions of the electromagnetic spectrum where the chloroplasts' antenna pigment usually don't capture light. In this way the assembly of SWCNTs within plants could be used to increase the amount of solar energy absorbed by the plants itself increasing the photosynthetic function of chloroplasts. Moreover, SWCNTs have been used as vector to localize useful nanoparticles for the oxygen production within the envelope and on the thylakoid of chloroplasts. In details, cerium nanoparticles, which have the ability to reduce ROS generation (Asati et al., 2010), preventing the consequential damage of pigments increasing the photosynthetic process have been localized in these specific regions. These objectives have constituted a plant nanobionics approach for plant biotechnology research: the contribution that molecular switches could give to this field is highly challenging. The bio applications of hybrid materials composed by carbon based structures and azobenzenes have been less explored if compared with spiropyrans. Guo and collaborators have proposed an innovative light-switchable SWCNTs hybrid device with potential applications as biomedicine sensor (Guo et al., 2013). They have prepared an hybrid system using the reversible interaction between a polymer containing an azobenzene moiety and a cyclodextrin (CD) containing SWCNTs through host-guest interaction between the AB and the CD. The assembly-disassembly of this system can be easily and reversibly controlled by light. The ternary system is well dispersed in water evidencing a strong interaction between the *trans* azobenzene isomer and the cyclodextrin. When the system is exposed to UV irradiation, the azobenzene reverts to its *cis* isomer, which presents only a weak interaction with CD causing a minor solubility and a precipitation of the system in the solvent. The mesostructured system has been deeply analyzed with transmittance measurements and UV-Vis and NIR spectroscopies that confirm the reversibility of the process. The ternary system has been used to prepare a transparent conducting film device opening the way to potential application in biomedicine. With this overview on the recent biological applications of photo responsive carbon nanomaterials (see Table 2) based on photochromic phenomena is really clear how this area of study presents good potentialities to be implemented, in particular the reversible photo isomerization behavior of AB and SP, should be chosen in the future applications because its reversibility, indeed compared to other molecules where to obtain the photo activation the process result in an irreversible modification of the chemicals structure (Klán et al., 2013), with AB and SP the materials prepared could present the additional properties of reversibility and cyclability that constitute a limitation in other systems (Yang et al., 2017).

**Table 2:** Hybrid photoresponsive materials and their applications.

**Materials**	**Applications**	**References**
GO/SP	Sensing of ions in cells: selective detection of fluoride ions in bovine serum	Li et al., 2013
SWCNTs/SP	Sensing of ions in cells: glassy carbon electrode to detect fluoride ions	Tao et al., 2016
rGO/SP	Cellular imaging: cancer detection in liver, kidney, spleen, lung for *in vivo* studies	Al Nahain et al., 2013
rGO/SP	Drug delivery: doxorubicin delivery in mice	Al Nahain et al., 2013
GO/SP-BODIPY	Bio-imaging: assembly of multicolor stimuli responsive nanoparticles for *in vitro* and *in vivo* studies	Sharker et al., 2014
SWCNTs/SP	Release of ions: light controlled release of anti-inflammatory zinc ion for *in vitro* studies	Del Canto et al., 2012
SWCNTs/SP	Electrochemical sensors: preparation of electrochemical switch for the identification of β-galactosidase	Tao et al., 2014
MWCNTs/SP	Control of enzyme activity: hybrid system to control the activity of the horseadish peroxidase	Song et al., 2011
graphene/SP	Control of enzyme activity: programmable bio-electrodes assembled with polymers conjugated systems	Parlak et al., 2016a,b
SWCNTs/AB	Biosensors: conducting film devices based on host-guest interaction between polymers and cyclodextrins	Guo et al., 2013

In the last years both SWCNTs and graphene have been deeply investigated in hybrid materials obtained from the assembly with light-responsive polymers: these composite materials have shown many interesting properties. Lemasson et al. have prepared a stable suspensions of SWCNTs in organic solvents by using a polymer with a fluorene backbone and an o-nitrobenzylether photocleavable unit (Lemasson et al., 2011). Several polymerization ratios between the fluorophore and the photocleavable unit have been investigated. When these systems are exposed to photo irradiation the suspension of functionalized SWCNTs precipitates due to the photocleavage of the nitrobenzyl subunit: the time scale of such process depends on the degree of polymer functionalization and its composition. The properties of this system can be improved by assembling AB or SP subunits in the polymer backbone (Imin et al., 2012). The photoswitchable polymer allows the preparation of composite nanomaterials that can be selectively dispersed in different solvents by switching the conformation of the molecule from *trans* to *cis* upon UV light irradiation. Li et al. have prepared hybrid CNT composites by assembly an epoxy resins, based on thermo-reversible Diels Alder network on NH_2_-functionalized CNTs (Li et al., 2017). By exposing the composite material to near-infrared radiation, a photothermal conversion of the light to thermal energy is observed. This system has been fully characterized and has shown the complete recyclability of the resin. Moreover, by regulating the power of the NIR laser, the photo-thermal conversion can be controlled. Graphene oxide mesostructures assembled with polymers have been proposed as anticancer platforms for chemo-phototherapy (Tran et al., 2015). First of all, the anticancer drugs doxorubicin and irinotecan have been loaded on the graphene functionalized with photoresponsive polymers (poloxamer 188). The mesostructured materials have allowed the delivery of the drugs close to cancer cells and their release upon irradiation with near-infrared laser. This new platform has been tested in terms of cellular uptake, cytotoxicity and photothermal efficacy also in resistant cancer cells' lines, presenting a very efficient scaffold which combine chemotherapy and photothermal therapy. In future the possibility to integrate these new hybrid systems by combining thermo or light controllable polymers with ABs and SPs subunits in the polymer structure and carbon nano materials can result in novel smart tools for several biological applications.

## Conclusions

The coupling of carbon nanomaterials, such as graphene and carbon nanotubes, with photo-switchable molecules, such as azobenzenes and spiropyrans, meets the growing and challenging requirements for biocompatible multifunctional systems. Novel hybrid nanomaterials for biological purposes should respond to a fundamental criterion: the biocompatibility of the new system must be preserved. Among the carbon nanomaterials both CNTs and graphene have evidenced good features in terms of biocompatibility and low toxicity. Progress in our ability to integrate these nanomaterials with molecular switches is needed in anticipation of future biomedical applications, particularly for the effective remote control of a biological function. The capability of azobenzenes and spiropyrans to response to several external stimuli, such as precise light's wavelength, pH, temperature, and coordination with anions or cations, in a rapid and reversible way as well as the different properties of their isomers have evidenced the potentials of these systems for the development of the next generation of smart bio nanomaterials. From the synthetic viewpoint both azobenzenes and spiropyrans can be synthetized with the desired features in order to obtained molecules with: (i) precise time-scale responsiveness to the external stimuli, (ii) the required solubility properties in the desired media and (iii) the specific functional groups for the conjugation to nanomaterials. Moreover, the possibility to chemically functionalize the surface of carbon nanomaterial, by using covalent and noncovalent approaches, allows the preparation of photo-switchable hybrid materials with well-defined and tunable physical and chemical properties, making those materials very promising scaffolds for biological applications. The successful applications of these systems in drug delivery, bioimaging and biosensors, which have been shown *in vitro* and *in vivo* studies, demonstrate the versatility of these materials and hold promises for their investigations in more complex systems, including their applications for the detection of biomarkers as noveldiagnostic techniques or as multi analytical nanodevices. The successful biomedical applications of bio-hybrid systems obtained from the conjugation of carbon based material with a variety of therapeutic biomolecules (Baker et al., 2002; Huang et al., 2002; Malarkey and Parpura, 2007; Nunes et al., 2010), from proteins to nucleic acids, can be widen by modulating their properties with light-responsive switches. On the other hand, the applications of photo responsive carbon nanomaterials in the vegetal world, where light has a dominant feature, could open novel perspectives toward the emerging nanobionics engineering. In order to deliver on their promises in biological systems, several improvements must be achieved to optimize these hybrid nanomaterials in order to obtain low aggregation, to maintain their individual nano-sized dimensions, and desired chemical and physical properties to allow biological's membrane permeability. New methodologies for the preparation of composites materials at different length scale from the sub-nanometers to the hundreds of micrometers scale must be investigated in order to obtain safe materials that must not be recognized by the immune systems of the species investigated. Further investigations should be performed to prepare materials that could be easily eliminated when they've explicated their function, including the study of metabolism, self-assembly in their environment of utilization as well as their clearance. To summarize, a greater attention should be given to deeply understand the mechanisms that control the dynamic properties of these materials in the biological context. There is a long way to go to exploit the properties and the safety of these smart nanomaterials. The realization of such safe materials will pave effectively the way to new responsive nanobiotechnologies.

## Author contributions

All authors listed have made a substantial, direct and intellectual contribution to the work, and approved it for publication.

### Conflict of interest statement

The authors declare that the research was conducted in the absence of any commercial or financial relationships that could be construed as a potential conflict of interest.
